# Investigation of *HLA-B* –21 M/T Dimorphism and Its Potential Role in COVID-19

**DOI:** 10.3390/ijms26136419

**Published:** 2025-07-03

**Authors:** David Martín-Rodríguez, Juan Francisco Gutiérrez-Bautista, Mónica Bernal, Antonio Rodriguez-Nicolas, José Ramón Vílchez, Ana Marín-Sánchez, Antonio Rosales-Castillo, Juan Sainz, Antonio José Cabrera-Serrano, Jorge Ceron-Hernandez, Miguel Ángel López-Nevot, Francisco Ruiz-Cabello, Pilar Jiménez

**Affiliations:** 1 Servicio de Análisis Clínicos e Inmunología, University Hospital Virgen de las Nieves, 18014 Granada, Spain; davidmar2003@correo.ugr.es (D.M.-R.); monica.bernal.sspa@juntadeandalucia.es (M.B.); joser.vilchez.sspa@juntadeandalucia.es (J.R.V.); anam.marin.sanchez.sspa@juntadeandalucia.es (A.M.-S.); manevot@ugr.es (M.Á.L.-N.); fruizc@ugr.es (F.R.-C.); mpilar.jimenez.sspa@juntadeandalucia.es (P.J.); 2 Departamento de Bioquímica, Biología Molecular e Inmunología III, University of Granada, 18016 Granada, Spain; 3 Instituto de Investigación Biosanitaria de Granada (ibs.GRANADA), 18012 Granada, Spain; 4 Laboratorio de Inmunología, Servicio de Hematología, Hospital Regional Universitario Carlos Haya, 29010 Malaga, Spain; antoniorn87@gmail.com; 5 Servicio de Medicina Interna, Hospital Universitario Virgen de Las Nieves, 18014 Granada, Spain; antonio.rosales.castillo.sspa@juntadeandalucia.es; 6 Genomic Oncology Area, GENYO, Centre for Genomics and Oncological Research: Pfizer/University of Granada/Andalusian Regional Government, PTS, 18016 Granada, Spain; juan.sainz@genyo.es (J.S.); antonio.cabrera@genyo.es (A.J.C.-S.); jorge.ceron@genyo.es (J.C.-H.); 7 Department of Biochemistry and Molecular Biology I, Faculty of Sciences, University of Granada, 18012 Granada, Spain

**Keywords:** HLA-E, COVID-19, NK cells

## Abstract

Natural killer (NK) cells play a key role in the innate immune response against viral infections. Their activity is regulated by a balance of activating and inhibitory signals, which are modulated by interactions with HLA class I molecules, including HLA-E. The *HLA-B* 21M/T dimorphism influences the availability of HLA-B leader peptides that stabilize HLA-E expression and modulate NK cell function via the NKG2A/CD94 receptor. To investigate the association between the *HLA-B* –21M/T dimorphism and the clinical severity of COVID-19, we analyzed a cohort of hospitalized patients with primary SARS-CoV-2 infection, who were genotyped for the *HLA-B* –21M/T dimorphism. Clinical data, lymphocyte counts, the neutrophil-to-lymphocyte ratio (NLR), and inflammatory markers were compared across genotypes. Contrary to previous studies suggesting a protective effect of the M/M genotype, we found no significant association between the *HLA-B* –21M/T dimorphism and COVID-19 severity, lymphocyte parameters, or inflammatory biomarkers. Our findings do not support a role for the *HLA-B* –21M/T dimorphism in modulating COVID-19 outcomes. These results underscore the complexity of NK cell regulation and highlight the need for integrative studies combining genetic, immunological, and functional data to better understand host factors influencing disease progression.

## 1. Introduction

The disease caused by the SARS-CoV-2 virus can present with phenotypes of varying severity, which are influenced by the patient’s clinical characteristics, the presence of comorbidities, and the individual’s genetic background. Numerous studies have identified genetic variants associated with susceptibility to infection or disease severity. Some genetic variants are associated with proteins involved in viral entry and mucosal function, such as *SLC6A20*, *SFTPD*, *ACE2*, and *TMPRSS2* [1,2]. Other variants directly affect components of the immune system, including polymorphisms in *IFNAR2*, *JAK1*, *TYK2*, and *TLR7*, among others [2,3]. HLA (human leukocyte antigen) molecules have also been identified as key genetic factors, given their crucial role in the development of adaptive immune responses. Numerous studies have reported associations between HLA alleles and either disease severity or susceptibility to infection. For example, the HLA-A*11:01 and HLA-C*04:01 alleles have been linked to severe disease [4], whereas the HLA-DRB1*04:01 allele has been associated with protection against severe COVID-19 [5]. However, other studies have found no significant associations in the populations analyzed [6].

HLA molecules are essential not only for antigen presentation and the development of adaptive immune responses but also for the regulation of natural killer (NK) cell activity. Specific HLA epitopes, such as Bw4/Bw6 and C1/C2, are recognized by NK cell receptors and modulate their activation [7]. In addition, HLA-B molecules exhibit a dimorphism in their leader peptide characterized by either a methionine (M) or a threonine (T) at position –21, which influences peptide binding to HLA-E [8]. HLA-E, a non-classical class I molecule, presents these peptides at the cell surface and interacts with the inhibitory receptor CD94:NKG2A on NK cells, delivering signals that prevent attacks on healthy cells. The presence of methionine (–21 M) leads to more efficient HLA-E stabilization and promotes NK cell education and functional maturation, whereas threonine (–21T) results in reduced HLA-E surface expression and less efficient NK cell education [9]. This dimorphism has been associated with modulation of NK cell responses in both antitumor and antiviral immunity [10,11], underscoring the importance of HLA-E-mediated sensing of cellular health in the immune response.

A recent study by Strunz et al. provided evidence suggesting a potential role of the *HLA-B* –21 M/T dimorphism in modulating COVID-19 severity [12]. In a cohort of 230 unvaccinated hospitalized patients, the authors observed that the M/M genotype was overrepresented among moderate cases and underrepresented in those with severe diseases. Using an age- and sex-matched analysis, they concluded that individuals carrying the M/M genotype exhibited less need for mechanical respiratory support and a more favorable clinical profile, including higher levels of IFN-γ. These findings support a possible protective role of the M/M genotype, potentially mediated by enhanced functionality of NKG2A+ NK cells [12]. However, due to the limited number of M/M individuals (*n* = 8), further validation in larger and more diverse cohorts is warranted.

In the present study, we investigated the role of the **HLA-B** –21 dimorphism in a cohort of 449 patients hospitalized with COVID-19, aiming to assess its potential influence on the risk of developing severe disease. Although previous studies have suggested that the M/M genotype might be associated with a more favorable clinical course [12], our results did not reveal any significant association between this polymorphism and COVID-19 severity. Given that the *HLA-B* –21 dimorphism affects HLA-E surface expression and NK cell education, further research is warranted to explore its potential role not only in COVID-19 but also in other infectious diseases. Our findings underscore the complexity of the genetic and immunological factors influencing disease outcomes and highlight the need for continued investigation into additional mechanisms underlying the clinical heterogeneity observed in SARS-CoV-2 infection.

## 2. Results

**Figure 1:**
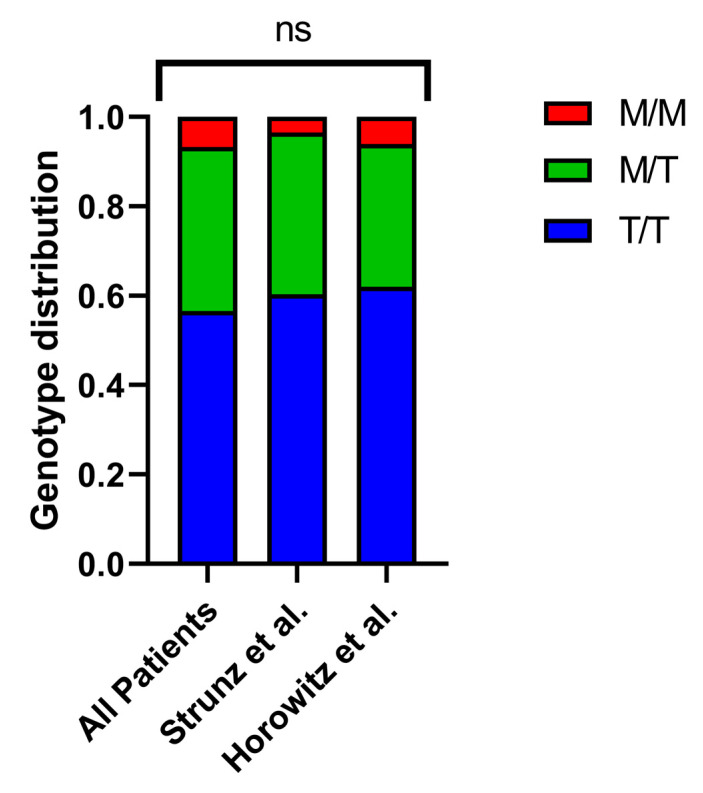
Genotype distribution of *HLA-B* –21 M/T dimorphism across patient cohorts. The distribution of M/M, M/T, and T/T genotypes in our study cohort and two reference populations—Strunz et al. (*n* = 230) [9] and Horowitz et al. (*n* = 8192) [12]—showed no significant differences.

To investigate how the *HLA-B* –21 M/T dimorphism might impact the severity of COVID-19, we analyzed the *HLA-B* alleles in a group of 449 patients hospitalized between April 2020 and January 2021. Then, we used imputation to analyze the *HLA-B* –21 M/T dimorphism (Supplementary Table S1) and categorized the patients into M/M, M/T, and T/T genotype groups. The distribution indicated that 6.7% of patients carried the M/M genotype, 36.7% had M/T, and 56.6% had T/T, which is consistent with previously documented frequencies (Figure 1) [9,12]. Additionally, we analyzed the age and sex distribution across the different groups and found no statistically significant differences.

**Figure 2:**
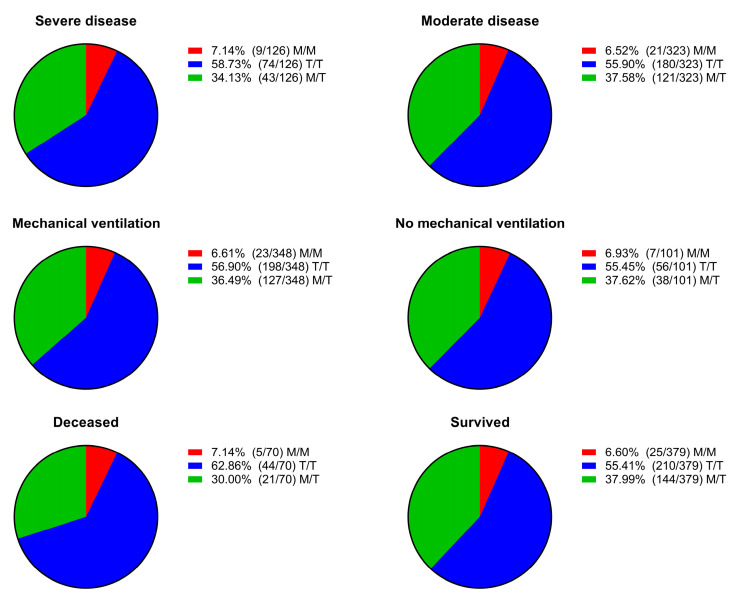
Distribution of genotypes (M/M, T/T, and M/T) according to clinical dichotomizations: disease severity (severe vs. moderate), need for mechanical ventilation (yes vs. no), and outcome (deceased vs. survived). No statistically significant differences were observed in genotype distribution among the compared groups.

We dichotomized the patients based on disease severity, need for mechanical respiratory support, and outcome. Then, we assessed the distribution of genotypes across the different dichotomized groups and found no statistically significant differences between them (Figure 2).

**Figure 3:**
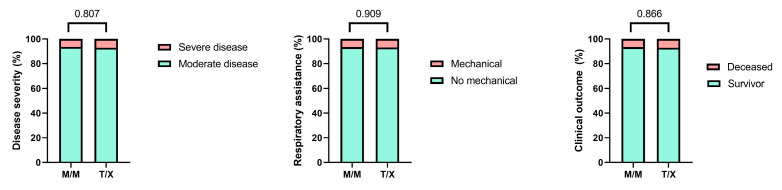
Comparison of clinical parameters between patients with M/M genotypes and those with T/X genotypes (M/T or T/T). Bars represent the percentage of patients according to disease severity (severe vs. moderate), need for mechanical respiratory support (mechanical vs. no mechanical), and clinical outcome (deceased vs. survivor). No statistically significant differences were observed between groups in any of the comparisons, as indicated by the corresponding *p*-values.

Given that the presence of the M variant in the *HLA-B* –21 dimorphism has been associated with enhanced NK cell activity, we classified patients into two groups: those carrying the M/M genotype and those carrying at least one T allele (M/T and T/T genotypes, grouped as T/X) [9]. We compared the genotype frequencies between these groups and did not observe any statistically significant differences (Figure 3).

Furthermore, a multivariate logistic regression analysis that adjusted for multiple comorbidities showed that the *HLA-B* –21 M/M genotype was not significantly associated with severe COVID-19 (adjusted OR = 2.15, p = 0.244) (Table 1). Among the comorbidities, asthma showed a trend toward association (aOR = 3.385, *p* = 0.082), although it did not reach statistical significance. Sex was the only variable that showed a statistically significant association with disease severity (aOR = 4.037, *p* = 0.004). Several variables with extremely wide confidence intervals or Exp(B) = 0 were excluded from interpretation due to low event counts.

**Figure 4:**
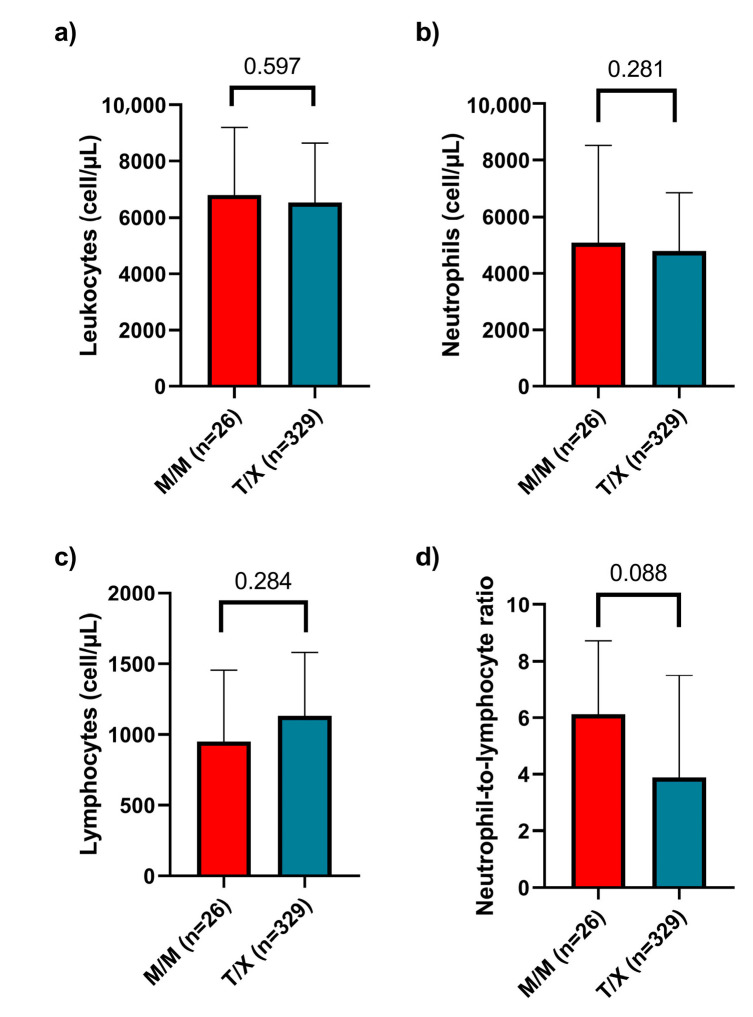
Leukocyte, neutrophil, and lymphocyte counts and neutrophil-to-lymphocyte ratio in patients with M/M versus T/X genotypes. (**a**) Total leukocyte count, (**b**) neutrophil count, (**c**) lymphocyte count, and (**d**) neutrophil-to-lymphocyte ratio were analyzed. No statistically significant differences were observed between the groups in any of the parameters evaluated. Data are presented as median and interquartile range (IQR).

Finally, we analyzed various clinical variables related to disease severity and inflammatory status. We examined leukocyte, neutrophil, and lymphocyte counts and the neutrophil-to-lymphocyte ratio (NLR) across different genotypes, but no statistically significant differences were observed. Additionally, we compared these parameters between the M/M and T/X genotype groups and similarly found no significant differences (Figure 4).

**Figure 5:**
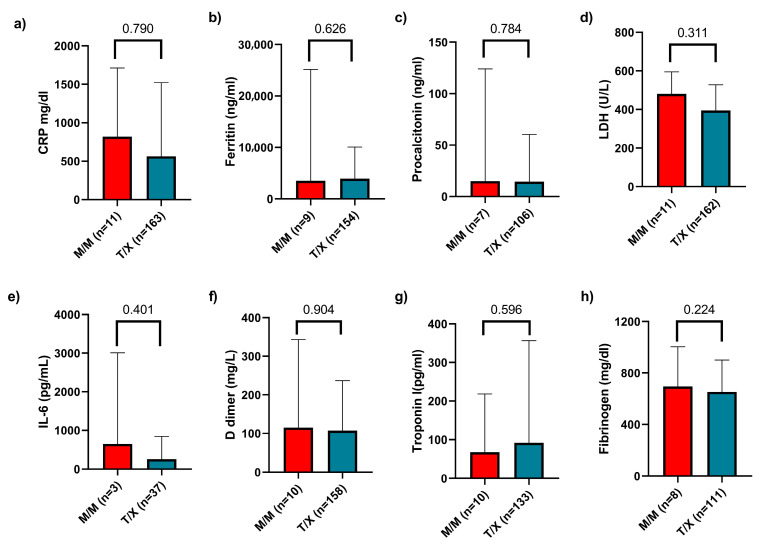
Comparison of biochemical inflammatory markers between M/M and T/X genotypes. Levels of (**a**) C-reactive protein (CRP), (**b**) ferritin, (**c**) procalcitonin, (**d**) lactate dehydrogenase (LDH), (**e**) interleukin-6 (IL-6), (**f**) D-dimer, (**g**) troponin I, and (**h**) fibrinogen were assessed. No statistically significant differences were observed between the two groups. Data are presented as median and interquartile range (IQR).

Regarding biochemical parameters, we collected data on C-reactive protein (CRP), ferritin, procalcitonin, lactate dehydrogenase (LDH), interleukin-6 (IL-6), D-dimer, troponin I, and fibrinogen levels. We compared these values across the three genotypes and found no statistically significant differences. Finally, a comparison was also made between the M/M and T/X groups, with no significant differences observed (Figure 5). Additionally, we compared these inflammatory markers between patients with mild versus severe disease and generally observed higher levels of inflammation in those with severe disease (Supplementary Table S2).

Finally, given the physical proximity between the HLA-B and HLA-C loci, we performed a linkage disequilibrium (LD) analysis between the *HLA-B* −21M dimorphism and the HLA-C1/C2 groups. The results showed a weak but positive LD between −21M and C1 (D = 0.014, D′ = 0.131, r^2^ = 0.0043) and a corresponding weak negative LD between −21M and C2 (D = −0.014, D′ = −0.131, r^2^ = 0.0043). These findings suggest a slight tendency for HLA-B −21M to co-occur with HLA-C1, in line with previous reports, although the very low r^2^ values indicate a negligible level of correlation between these loci in our population. Overall, there is no evidence of strong linkage disequilibrium between the *HLA-B* −21 polymorphism and HLA-C ligand groups.

Additionally, we analyzed the potential association between *HLA-C* genotype (C1/C1, C1/C2, C2/C2) and disease severity. Genotypic frequencies were compared between patients with mild and severe forms of COVID-19. However, no statistically significant association was observed between the C1/C2 groups and clinical severity, suggesting that, in our cohort, *HLA-C* allele variation is not related to disease progression.

## 3. Discussion

Innate immunity plays a fundamental role in regulating the course of COVID-19, enabling rapid infection control, as previously demonstrated for type I interferon responses. Among innate immune cells, natural killer (NK) cells are critical effectors in the early defense against viral infections. In this context, we analyzed the *HLA-B* –21 M/T dimorphism in a cohort of patients with primary SARS-CoV-2 infection who required hospitalization. This dimorphism influences the binding affinity of the HLA-B leader peptide to HLA-E molecules, which determines HLA-E surface expression. Membrane expression of HLA-E is essential for its recognition by NK cells via the inhibitory receptor NKG2A/CD94, thereby modulating NK cell activity through activation or inhibition [13]. It has been described that certain viruses have evolved mechanisms to exploit HLA-E–mediated immune regulation to evade NK cell responses. For example, human cytomegalovirus (HCMV) encodes UL40 glycoproteins that mimic HLA class I leader peptides, stabilizing HLA-E on the surface of infected cells and enhancing inhibitory engagement with NKG2A, thereby impairing NK cell-mediated cytotoxicity [14]. A similar immune evasion strategy has been reported for Epstein–Barr virus (EBV) via its LMP-1 protein [15].

The influence of the *HLA-B* –21 M/T dimorphism has also been investigated in the context of other viral infections, such as HIV. Although its exact implications remain to be fully elucidated, some studies suggest that individuals with the M/M genotype may experience faster disease progression and a reduced capacity to clear infected cells, potentially due to increased inhibitory signaling mediated by the NKG2A receptor [11,16]. Furthermore, research by Cubero et al. indicates that HIV/HCMV co-infection can modify HLA-E ligand presentation, promoting the expansion of NKG2C^+^ NK cell subsets [17]. In contrast, a study by Strunz et al. reported that individuals with the M/M genotype exhibit less severe clinical trajectories in COVID-19 [12]. Additionally, other reports have described enhanced functional activity of NKG2A^+^ NK cells in M/M individuals [9], as well as effective NK cell-mediated responses against SARS-CoV-2–infected cells in this genotype group [18]. However, in our study, which included a larger patient cohort, we did not observe a significant association between the M/M genotype and a milder form of COVID-19.

In our study, we analyzed the potential association between the *HLA-B* –21M/T dimorphism and COVID-19 severity. In contrast to the findings of Strunz et al., who reported a correlation between the M/M genotype and milder disease, we did not observe significant differences in clinical severity based on genotype. These results may appear contradictory to those reported in other viral infections [11,16]. The presence of the M/M genotype is associated with increased surface expression of HLA-E, which may lead to reduced activation of NK cell cytotoxic function through enhanced engagement of the inhibitory receptor NKG2A. This could facilitate greater viral replication and dissemination. On the other hand, reduced NK cell activation may also result in decreased production of proinflammatory cytokines such as interferon-gamma (IFN-γ), tumor necrosis factor-alpha (TNF-α), and granulocyte-macrophage colony-stimulating factor (GM-CSF) [19]. This immunomodulatory effect could potentially prevent the onset of cytokine storm and the development of severe COVID-19 [20], thereby supporting the findings reported by Strunz et al.

A potential explanation for the findings reported by Strunz et al. could be the presence of LD between HLA-B −21M alleles and HLA-C2 group alleles, which has been previously described in Caucasian populations but is less frequent in individuals of African ancestry [17]. Such LD may be stronger in Northern European populations, including the Swedish cohort studied by Strunz et al., than in Southern European populations like ours. In our study, we evaluated this possibility by performing a linkage disequilibrium analysis between the *HLA-B* −21M polymorphism and HLA-C1/C2 groups. The results showed only weak LD: a slight positive association between −21M and C1 and a corresponding weak negative association between −21M and C2. These values indicate negligible correlation between these loci in our cohort. Furthermore, we found no significant association between HLA-C genotypes and COVID-19 severity. Altogether, these findings suggest that, in our Mediterranean population, the effect of the *HLA-B* −21M allele is not confounded by linkage to HLA-C2 alleles and that differences in LD structure may underlie discrepancies observed across studies from different populations.

As is well known, yet still challenging to fully elucidate, NK cell activity is precisely regulated by a balance between activating and inhibitory signals mediated by specific membrane receptors that recognize HLA molecules, among others [20,21]. Therefore, NK cell activation or inhibition must be understood as the result of a complex network of interactions, rather than through the analysis of individual receptors in isolation. For instance, in our previous work, we reported an association between the A*9 STR polymorphism in the *MICA* gene—an activating ligand for NK cells—and both SARS-CoV-2 infection and the development of symptomatic COVID-19 [22]. Moreover, deletion of the *KLRC2* gene, which encodes the activating receptor NKG2C, has been linked to increased susceptibility to severe forms of COVID-19. Similar associations have been reported for certain variants of the HLA-E*0101 allele [23]. Regarding KIR gene polymorphisms, the combination of the activating receptor KIR2DS2 with the HLA-C1 allele has been associated with reduced mortality in COVID-19 patients, whereas increased expression of the inhibitory receptor KIR2DL1 has been observed in individuals with severe disease [24,25]. Finally, patients with severe COVID-19 consistently show elevated expression of the inhibitory receptor NKG2A on NK cells, which is associated with an exhausted phenotype and impaired immune responses [26]. Given the wide range of findings concerning NK cell activation and COVID-19 severity, there is a clear need for further studies that assess, from an integrative perspective, the role of NK cells and the relevance of their activating and inhibitory receptors, along with their respective ligands.

In contrast to the cohort analyzed by Strunz et al., where approximately 50% of the patients required mechanical ventilation, this proportion was lower in our study (~20%). Several factors may account for this difference, including a broader clinical profile in our cohort that encompassed hospitalized patients with moderate symptoms, as well as possible differences in clinical thresholds for initiating ventilatory support. In our setting, the decision to initiate mechanical ventilation (invasive or non-invasive) was based on clinical judgment and guided by physiological parameters such as peripheral oxygen saturation, respiratory rate, and the arterial oxygen partial pressure to inspired oxygen fraction ratio (PaO_2_/FiO_2_, or PaFi), in line with routine clinical practice. Additionally, resource limitations during peak periods of the pandemic may have contributed to stricter criteria for initiating ventilatory support. Nevertheless, the definition of severe disease used in our study was based on objective indicators of organ dysfunction (ventilation, ECMO, ARDS, shock, or multiorgan failure), ensuring the robustness of the severity classification. In addition to evaluating the role of the *HLA-B* –21 M/T dimorphism, we performed a multivariate logistic regression analysis including a range of clinical and demographic variables. Interestingly, sex emerged as the only statistically significant predictor of severe COVID-19, with males exhibiting a markedly higher risk (adjusted OR = 4.037, *p* = 0.004). This finding aligns with multiple studies, including a global meta-analysis, which reported significantly higher rates of ICU admission and mortality among men [27]. These differences have been attributed to sex-based immune regulation, hormonal influences, and distinct comorbidity profiles. Other variables in our analysis, such as asthma and liver disease, showed a trend toward association with severe outcomes but did not reach statistical significance. Furthermore, the absence of significant differences in the frequency of the –21 M/M genotype among deceased patients—a group unlikely to be affected by potential underestimation of disease severity—further supports the strength of our findings.

To further evaluate the clinical impact of the polymorphism, we compared lymphopenia, neutropenia, and the NLR but found no significant differences between individuals carrying the M/M genotype and the rest of the cohort. However, Strunz et al. reported an association between the M/M genotype and lower levels of lymphopenia and NLR, suggesting a milder disease course in these patients [12]. Additionally, we analyzed several inflammatory biochemical parameters and found no significant differences among the different genotypes studied. This discrepancy could be attributed to variations in the immunological background of the patient populations, the timing of laboratory data collection, the stage of infection, or the influence of other genetic factors modulating the immune response.

In conclusion, our findings do not support an association between the *HLA-B* –21 M/T dimorphism and COVID-19 severity, in contrast to previous reports. Nevertheless, this does not exclude the potential contribution of other HLA polymorphisms to the clinical course of the disease. Our results, obtained from a larger and well-characterized patient cohort, highlight the complexity of NK cell-mediated immune responses and the importance of considering multiple genetic and immunological factors to evaluate disease outcomes. One limitation of our study is the relatively small size of the *HLA-B* –21 M/M subgroup, which represented only about 6.7% of the cohort. This may have reduced the statistical power to detect modest associations and should be taken into account when interpreting our findings. Future studies should aim to integrate functional assays of NK cell activity, broader HLA and KIR genotyping, and longitudinal clinical follow-up in order to better delineate the immunogenetic factors that influence susceptibility to severe COVID-19 and other viral infections.

## 4. Methods and Materials

### 4.1. Population of the Study

This study included a cohort of 449 patients hospitalized with COVID-19 at the Hospital Universitario Virgen de las Nieves in Granada, Spain. Sample collection took place between April 2020 and January 2021. Patients were eligible for inclusion if they required hospitalization due to pneumonia or respiratory complications related to COVID-19 confirmed by a positive SARS-CoV-2 PCR test. The participants included in the study had not received a SARS-CoV-2 vaccine. A summary of the patients’ clinical features and comorbid conditions is provided in Table 2. Patients with severe disease were defined as those who developed respiratory failure requiring invasive or non-invasive mechanical ventilation, extracorporeal membrane oxygenation (ECMO), acute respiratory distress syndrome (ARDS), shock, and/or multiorgan failure.

For the analysis of biochemical parameters, data were available for only 186 patients, whereas leukocyte, neutrophil, and lymphocyte counts were accessible for a total of 361 patients. These data were obtained at the time of hospital admission, prior to the administration of any treatment.

The study received ethical approval from the Portal de Ética de la Investigación Biomédica of the Junta de Andalucía (Ref. 0766-N-20). All participants gave their written informed consent prior to inclusion in the study.

Peripheral venous blood samples were collected from all participants, and genomic DNA was isolated using the QIAMP DNA Blood Mini Kit (Qiagen, Hilden, Germany) in accordance with the manufacturer’s protocol. High-resolution genotyping of HLA- B and -C loci was conducted utilizing the LABType assay based on sequence-specific oligonucleotide probes (One Lambda, Canoga Park, CA, USA). The procedure involved the PCR amplification of target regions using sequence-specific primers, followed by hybridization with allele-specific probes conjugated to fluorescent microspheres labeled with phycoerythrin. The fluorescence signal was analyzed on a LABScan 100 platform (Luminex xMAP, Austin, TX, USA), and HLA allele assignments were performed using HLA-Fusion software, version 4.6.0 (Palex Medical, Barcelona, Spain).

### 4.2. Genotyping Based on the HLA-B –21 M/T Dimorphism

The *HLA-B* –21 M/T dimorphism (corresponding to SNV rs1050458) was inferred following established methods [9,28] by classifying the genotype according to the predominant allele groups, as outlined in Supplementary Table S1.

### 4.3. C1/C2 Groups Analysis

The classification of *HLA-C* alleles into C1 and C2 groups was based on their amino acid at position 80, as previously described [29]. Each subject’s *HLA-C* genotype was analyzed and categorized as C1/C1, C1/C2 (heterozygous), or C2/C2, depending on the combination of alleles.

### 4.4. Statistical Analysis

Genotypic distribution comparisons were conducted using the chi-square test or Fisher’s exact test (two-tailed), depending on the data requirements, through contingency tables. To account for multiple comparisons, significance thresholds were adjusted using the Bonferroni correction. Risk associations were evaluated by calculating odds ratios (ORs) with 95% confidence intervals (CIs). Group differences in non-normally distributed variables—assessed via the Kolmogorov–Smirnov test—were analyzed using the Kruskal–Wallis or Mann–Whitney U tests. All statistical analyses were performed using SPSS software (version 26 for Windows; IBM Corp., Armonk, NY, USA).

Continuous non-parametric variables were reported as medians with interquartile ranges. The Mann–Whitney U test was applied for group comparisons in these cases. *p*-values were unadjusted, and a significance level of α = 0.05 was used to determine statistical significance.

Additionally, multivariate logistic regression analysis was conducted to assess the independent association between *HLA-B* –21 genotypes and COVID-19 severity, adjusting for relevant clinical comorbidities (e.g., hypertension, diabetes, obesity, and chronic pulmonary and hepatic conditions). Variables with very low prevalence or unstable estimates were excluded from the final model to ensure statistical validity. Adjusted odds ratios (aORs) and 95% confidence intervals were calculated. Model stability and potential multicollinearity were assessed. This analysis was also performed using SPSS software.

Linkage disequilibrium (LD) analysis between *HLA-B* −21 dimorphisms (M/T) and *HLA-C* C1/C2 groups was performed using Arlequin software version 3.5.2.2 (University of Bern, Bern, Switzerland). An input file was prepared in the Arlequin-specific format (.arp), including phased genotypic data for both loci. LD parameters, including D, D′ (normalized linkage disequilibrium), and r^2^ (squared correlation coefficient), were calculated using default settings, and results were interpreted to assess the degree of non-random association between alleles.

## Figures and Tables

**Table 1:** Multivariate logistic regression analysis of predictors of severe COVID-19.

Variable	*p*-Value	Exp(B)
Sex (Male)	0.004	4.037
Age	0.289	1.021
M/M Genotype	0.244	2.154
Asthma	0.082	3.385
Liver disease	0.098	15.326
Diabetes	0.585	1.341
Obesity	0.301	1.521
AH	0.157	1.998
Myocardial Infarction	0.822	0.832
Heart Failure	0.565	0.550
Hemiplegia	1	3.385
COPD	0.683	0.682
Gastric Ulcer	0.315	4.492
Non-metastatic Neoplasm	0.953	0.927
Lymphoma	0.676	2.143
CKD	0.292	0.316
Immunosuppression	0.946	0.919

AH: arterial hypertension; CKD: chronic kidney disease; COPD: chronic obstructive pulmonary disease. Exp(B): exponentiated coefficient from the logistic regression model, representing the odds ratio (OR). It indicates how much the odds of severe COVID-19 increase (if >1) or decrease (if <1) for a one-unit increase in the predictor variable.

**Table 2:** Clinical, demographic, and comorbidity profile of hospitalized COVID-19 patients.

Parameter	Value (*n* = 449)
Age (years)	Mean: 62 (range: 25–98)
Sex	
Female	205 (45.5%)
Male	244 (54.5%)
ICU admission	126 (26.1%)
Non-ICU hospitalization	323 (73.9%)
Required mechanical ventilation	101 (20.9%)
No mechanical ventilation	348 (79.1%)
Outcome	
Deceased	70 (14.5%)
Survived	379 (85.5%)
**Prevalence of Comorbidities Among Hospitalized Patients**	
**Comorbidity**	***n* (%)**
Hypertension	196 (43.6%)
Diabetes mellitus (DM)	101 (22.4%)
Overweight/obesity	72 (16%)
Chronic kidney disease (CKD)	33 (7.3%)
Chronic obstructive pulmonary disease (COPD)	32 (7.1%)
Asthma	26 (5.8%)
Myocardial infarction (MI)	26 (5.8%)
Heart failure (HF)	25 (5.5%)
Cerebrovascular disease (CVD)	22 (4.9%)
Peripheral arterial disease (PAD)	11 (2.4%)

DNA isolation and HLA-B genotyping procedures.

## Data Availability

The data presented in this study are available on request from the corresponding author. The data are not publicly available due to the fact that they correspond to HLA typing of patients.

## Supplementary Materials

The supporting information can be downloaded at https://www.mdpi.com/article/10.3390/ijms26136419/s1.
