# Supplementary material for: Investigation of HLA-B –21 M/T Dimorphism and Its Potential Role in COVID-19
Source: Int J Mol Sci. 2025 Jul 3;26(13):6419. doi: 10.3390/ijms26136419 (PMC12250186; doi:10.3390/ijms26136419)
Supplement: Supplementary file 1 [file ijms-26-06419-s001.zip › Supplementary Table S1.pdf]

Table S1. Distribution of the –21 M/T dimorphism (rs1050458) across HLA-B alleles.

**Allele Group**	**HLA-B –21 M/T Dimorphism**
HLA-B*07	M
HLA-B*08	M
HLA-B*13	T
HLA-B*14	M
HLA-B*15	T
HLA-B*18	T
HLA-B*27	T
HLA-B*35	T
HLA-B*37	T
HLA-B*38	M
HLA-B*39	M
HLA-B*40	T
HLA-B*41	T
HLA-B*42	M
HLA-B*44	T
HLA-B*45	T
HLA-B*46	T
HLA-B*47	T
HLA-B*48	M
HLA-B*49	T
HLA-B*50	T
HLA-B*51	T
HLA-B*52	T
HLA-B*53	T
HLA-B*54	T
HLA-B*55	T
HLA-B*56	T
HLA-B*57	T
HLA-B*58	T
HLA-B*59	T
HLA-B*67	M
HLA-B*73	M
HLA-B*78	T
HLA-B*81	M
HLA-B*82	T
HLA-B*83	T
