# Supplementary material for: Investigation of HLA-B –21 M/T Dimorphism and Its Potential Role in COVID-19
Source: Int J Mol Sci. 2025 Jul 3;26(13):6419. doi: 10.3390/ijms26136419 (PMC12250186; doi:10.3390/ijms26136419)
Supplement: Supplementary file 1 [file ijms-26-06419-s001.zip › Supplementary Table S2.pdf]

Table S2. Comparison of clinical laboratory parameters between moderate and severe COVID-19 cases

Clinical Laboratory Parameters	Moderate Disease	Severe Disease	P-value
Leukocyte (cells/µl)	6510 (3520)	7305 (4113)	0.272
Neutrophils (cells/µl)	4730 (3290)	6365 (5190)	0.061
Lymphocytes (cells/µl)	1150 (810)	855 (675)	**9E-6**
NLR	3.85 (4.23)	7.7 (14.1)	**2.2E-4**
CRP (mg/dL)	1677 (1137)	2935 (3315)	**0.003**
Ferritin (ng/mL)	14394 (16143)	3604 (5710)	0.265
Procalcitonin (ng/ml)	16 (28)	187 (649)	**1.1E-5**
LDH (U/L)	484 (217)	595 (309)	**0.001**
IL-6 (pg/mL)	366 (679)	973 (1769)	**0.007**
D-dimer (mg/L)	164 (221)	579 (1875)	**0.004**
Troponin I (pg/mL)	94 (260)	749 (828)	**2E-4**
Fibrinogen (mg/dL)	651(264)	818 (489)	**0.016**

Values are expressed as median with interquartile range (IQR) in parentheses. Bold values indicate statistical significance. CRP: C-reactive protein; IL-6: interleukin-6; LDH: lactate dehydrogenase; NLR: neutrophil-to-lymphocyte ratio.
